# RNA Editing Therapeutics: Advances, Challenges and Perspectives on Combating Heart Disease

**DOI:** 10.1007/s10557-022-07391-3

**Published:** 2022-10-14

**Authors:** Maria Birgaoanu, Marco Sachse, Aikaterini Gatsiou

**Affiliations:** 1 Vascular Biology and Medicine Theme, Faculty of Medical Sciences, Biosciences Institute, Newcastle University, International Centre for Life, Central Parkway, Newcastle Upon Tyne, NE1 3BZ UK; 2 Department of Cardiovascular Surgery, University Heart and Vascular Center, University Medical Center Hamburg-Eppendorf, University of Hamburg, Hamburg, Germany

**Keywords:** ADAR, APOBEC, RNA editing, RNA modifications, Gene expression, RNA therapeutics, Cardiovascular disease

## Abstract

Cardiovascular disease still remains the leading cause of morbidity and mortality worldwide. Current pharmacological or interventional treatments help to tackle symptoms and even reduce mortality, but cardiovascular disease cases continue to rise. The emergence of novel therapeutic strategies that precisely and efficiently combat cardiovascular disease is therefore deemed more essential than ever. RNA editing, the cell-intrinsic deamination of adenosine or cytidine RNA residues, changes the molecular identity of edited nucleotides, severely altering the fate of RNA molecules involved in key biological processes. The most common type of RNA editing is the deamination of adenosine residue to inosine (A-to-I), which is catalysed by adenosine deaminases acting on RNA (ADARs). Recent efforts have convincingly liaised RNA editing-based mechanisms to the pathophysiology of the cardiovascular system. In this review, we will briefly introduce the basic concepts of the RNA editing field of research. We will particularly focus our discussion on the therapeutic exploitation of RNA editing as a novel therapeutic tool as well as the future perspectives for its use in cardiovascular disease treatment.

## Introduction

Progress in the past two decades in molecular genetics, such as genome-wide association studies, has led to the identification of genes and variants responsible for human disease, including cardiovascular disease (CVD). Results from such studies have been used not only to determine the certain risk variants pose for disease development but also to predict drug efficiency. Despite this progress, many risk factors remain undetected. According to the World Health Organisation, cardiovascular disease complicated by heart attack and stroke represents 32% of all global death and remains the leading cause of death in 2022 [1]. It is estimated that 7.5% of the approximately 20,500 human protein-coding genes are directly involved in RNA metabolism [2]. Therefore, there is a need to explore beyond the “first level” of genetic information, that is DNA, and investigate how regulation and changes at the RNA level may account for CVD.

**Fig. 1:**
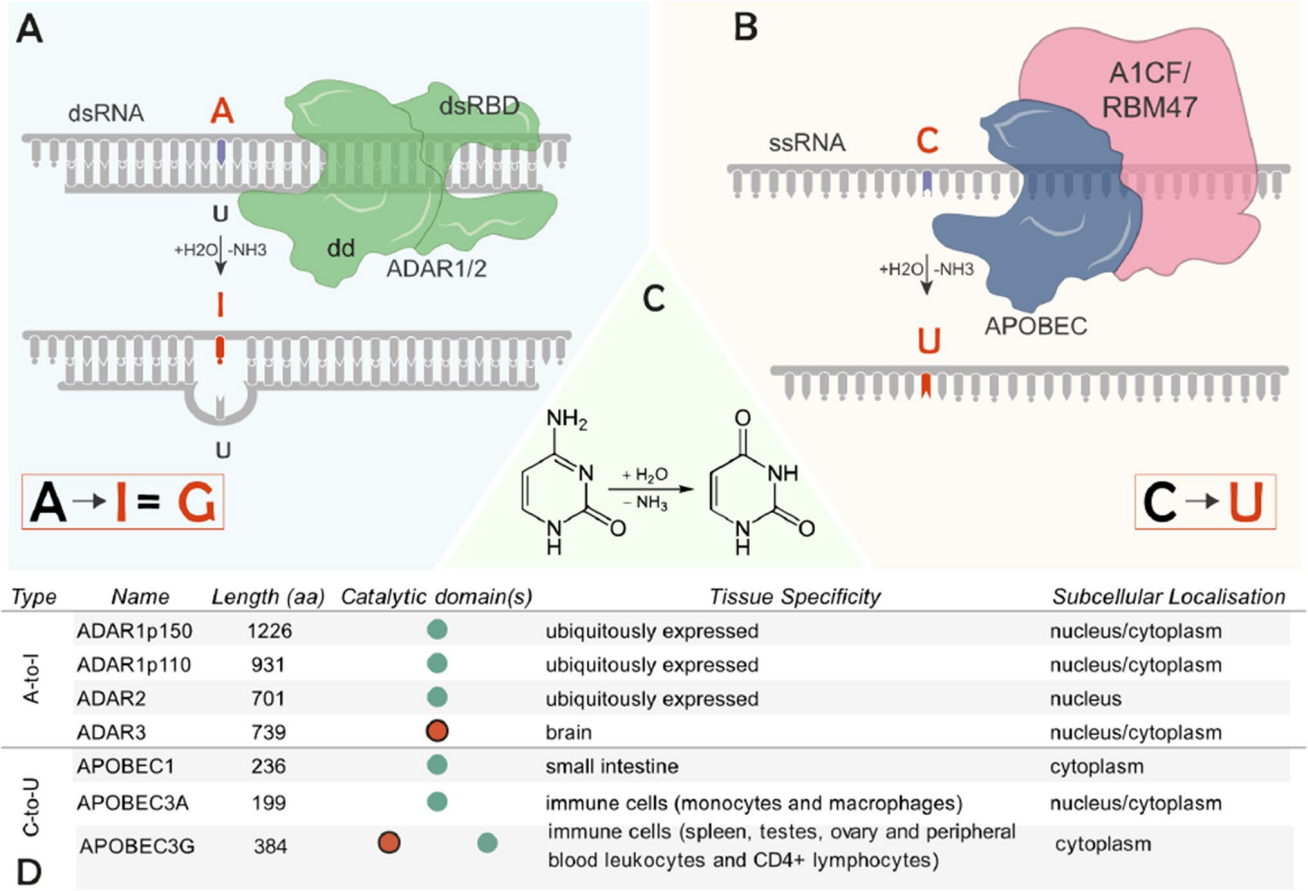
Mechanism of RNA editing and characterisation of ADARs/ APOBECs. **A** ADARs bind to dsRNAs and catalyse the deamination of adenosine residues to inosines, which is interpreted as guanosine by the translational and splicing machinery. **B** APOBECs bind to ssRNA and convert cytidine to uridine. APOBEC1 binds to two possible co-factors, A1CF or RBM47, both with different mRNA target sets. APOBEC3A and APOBEC3G are not known to require any such co-factors. **C** ADARs and APOBECs catalyse deamination by removing an amino group from adenosine and cytidine residues, respectively. **D** Characterisation of RNA base editors. Green (no outline) and red (with black outline) circles represent active and inactive catalytic domains, respectively. Tissue specificity and subcellular localisation information were sourced from Uniprot. ADAR, adenosine deaminases acting on RNA; APOBEC, apolipoprotein B mRNA editing catalytic polypeptide-like; dsRNA, double-stranded RNA; ssRNA, single-stranded RNA; dd, deaminase domain; dsRBD, dsRNA-binding domain; H_2_O, water; NH_3_, ammonia; O, oxygen; N, nitrogen

RNA metabolism is mainly controlled by RNA-binding proteins, and any iteration in this important process may disturb the outcome of gene expression flow. Adenosine deaminases acting on RNAs (ADARs) are double-strand RNA-binding proteins that chemically convert adenosine (A) bases within RNA molecules to inosine (I), with the latter further interpreted as guanosine by the cellular machineries (Fig. 1A) [3]. In humans, there are two catalytically active ADAR orthologs: ADAR1 and ADAR2 [4, 5]. Mammals have also a third but catalytically inactive ADAR, ADAR3, which appears to be predominantly expressed in the brain [6, 7]. ADARs have an N-terminal dsRNA-binding domain and a C-terminal deaminase domain responsible for deaminating adenosines. Although lacking motif enrichment at the primary sequence level, sequence context analysis has suggested that A-to-I editing often occurs at editing-enriched regions (EERs) [8], with 50 (upstream) and 30 (downstream) nearest base preferences as 50: U > A > C > G and 30: G > C = A > U for ADAR1 or 50: U > A > C > G and 30: G > C > U = A for ADAR2 [9]. Moreover, editing-enriched regions are often present within *Alu* elements within introns or the 3’UTR due to their ability to form double-stranded RNA [10, 11]. Inverted *Alu* elements forming dsRNA are the prerequisite target of ADAR-mediated RNA editing [10, 11], resulting in altering RNA metabolism [7]. Another type of RNA editing is the deamination of a cytosine (C) to uracil (U) base catalysed by apolipoprotein B editing catalytic enzymes (APOBECs) (Fig. 1B). Given that C-to-U RNA editing seems to be less commonly met along the transcriptome compared to A-to-I RNA editing, it will be only briefly mentioned in this review. The deamination reaction catalysed by ADARs and APOBECs is common (Fig. 1C**)**. However, the structure, tissue and subcellular localisation of A-to-I and C-to-U RNA base deaminases differ significantly (Fig. 1D).

The implication of editing by ADARs is that information encoded within DNA may be changed at the RNA level. We also know that all RNA types, not only mRNA, may be subjected to editing. As such, RNA base deaminases seem to have a complex role in the expression, function and stability of RNAs. ADAR1 is indispensable for life [4]. Ablation of *Adar1* in murine model embryos results in embryonic lethality owing to defective haematopoiesis and liver disintegration at E11.5–12.5. Specifically, further histological analysis of *Adar1* ablated embryos shows widespread cell death and ineffective haematopoiesis [12, 13]. ADAR1 has also recently piqued the interest of cardiovascular disease researchers, as A-to-I RNA editing has been found to be significantly elevated in patients with atherosclerosis and congenital heart defects [14, 15].

A well-known substrate of ADAR2 is the glutamate receptor *GluR2* mRNA. Under normal conditions, nearly 100% of the *GluR-B* mRNAs are edited at Q/R (glutamine/arginine) site-607 [5, 16]. Unedited *GluR-B* exhibits high Ca^2+^ permeability due to the negative charge of glutamine, which also leads to cell toxicity and death [5, 17]. Deletion of *Adar2* is postnatally lethal in mice as these mice suffer from seizures indicating severe neurological issues. However, *Adar2* deficient mice are rescued through A > G point mutations introduced in the genomic regions of Gria2 that are normally Adar2 editing sites to simulate the pre-edited condition, supporting the pivotal role of A-to-I RNA editing and ADAR2 for neuron homeostasis and overall mammalian physiology [18].

Site-directed RNA editing (SDRE) is an important set of tools that aims to harness the editing ability of A-to-I and C-to-U RNA base editors and guide them to specific RNA targets in order to correct pathogenic point mutations previously occurred in DNA at the RNA level. SDRE is highly programmable and aims to edit single nucleotides with precision. The SDRE methods discussed in this review can either be designed to recruit endogenously expressed ADARs to a specific mRNA target or engineered to contain a catalytic domain that performs deamination. Due to the transient nature of RNA, such therapeutic methods pose a much smaller clinical risk and ethical concern by not introducing permanent changes. In this review, we discuss the main changes that RNA editing affects in cardiovascular gene expression and biology, as well as we introduce the concept of SDRE tools, the challenges of their application and their perspectives about boasting a therapeutic option against cardiovascular disease.

## How RNA Editing May Affect RNA Metabolism and Cardiovascular Biology?

RNA editing is one of the most evolutionary conserved properties of RNAs, and it occurs in many living organisms, including humans [19–21]. We have previously summarised the first efforts which have linked RNA editing with cardiovascular pathophysiology in detail [4, 22]. RNA editing was first characterised in 1987, with the discovery that a C-to-U conversion within the sequence of apolipoprotein B (ApoB) introduced a termination codon, thereby resulting in a shorter ApoB isoform [23]. Since then, our knowledge of RNA editing has broadened, and we now know that all RNA types can be subjected to editing. In recent years, an increasing number of publications address how RNA modifications affect localisation [14, 24, 25], stability [14, 26–28] or activity of coding and non-coding RNAs [29, 30].

RNA editing is predominantly carried out by adenosine to inosine (A-to-I) editors, which are members of the ADAR protein families and cytidine to uridine (C-to-U) editors, which are members of the AID/APOBEC family [31]. There are multiple ways of expression and function of protein-coding genes that can be altered through mRNA editing. Most obviously, A-to-I and/or C-to-U conversions can alter the actual mature mRNA sequence since I (inosine) is recognised as G (guanosine) by the translational and splicing machinery [32]. Looking at the codon table, one can see how some of these changes might not have an impact on protein function, as they are synonymous (e.g., CUA(Leu) to CU(I)G(Leu)). RNA editing might also introduce readthrough mutations. For example, the STOP codon UAA may be edited to Trp UGG(II) by ADAR1 on the EGFP mRNA [33]. However, point mutations can also change the identity of amino acids, with missense mutations being some of the most common causes of genetic disease. This does not come as a surprise since amino acid interactions are crucial for protein structure, which in turn is crucial for protein function. Although the recoding effects of RNA editing with functional consequences are scarce in the literature, Michael Jantsch’s lab elegantly demonstrated the requirement of RNA editing on filamin A for the maintenance of normal blood pressure, especially during ageing, a major risk factor for cardiovascular disease [34]. Jantsch and colleagues led these pioneering efforts by generating mice lacking the dsRNA structure that is bound by ADARs, thus catalysing editing on filamin A pre-mRNA [34].The editing of filamin A has been previously shown to lead to a recoding event on its cognate protein, and filamin A is known for its pivotal role in protecting cardiovascular physiology [34].

Additionally, certain point mutations might introduce novel sites for RNA deaminases. Recently, A:C mismatches in RNA:DNA hybrids induced by point mutations at telomeric R-loops have been proven preferred targets of ADAR1p110 [35]. In this case, ADAR1p110-mediated editing leads to the formation of I:C matches, which facilitate the degradation of the RNA strand by RNase H2 [35, 36]. The ADAR1-mediated control of R-loops is necessary for cancer cells to evade replication stalling and promote their proliferation [37, 38]. The absence of RNA editing has also been linked to disease. For example, protein recoding through RNA editing has been found significantly decreased in Alzheimer’s disease in 22 different genes [39]. However, the relevance of this aspect for the cardiovascular disease remains yet elusive.

Admittedly, most RNA editing occurs within non-coding regions and especially within *Alu* regions [7, 40, 41]. We have previously reported that during atherosclerotic cardiovascular disease (ASCVD), ADAR1-mediated RNA editing is required for the expression of cathepsin S (CTSS), a matrix degradation enzyme with an important part in the development and progression of ASCVD [14, 42]. Editing of *Alu* elements, which are repetitive elements, within the 3’UTR of *CTSS* mRNA allowed RNA-binding protein (RBP) to the edited mRNA and thereby increased mRNA stability as well as CTSS expression [14]. As such, RNA editing of specific nucleotides across a hotspot of editing, the *Alu* region of CTSS, was strongly correlated with CTSS expression and cardiovascular disease clinical outcomes in the biomaterial of patients with different stages of ASCVD.

MicroRNAs are short non-coding RNA molecules that regulate the expression of protein-coding genes by complementarily base-pairing via their seed region to specific sequences within the 3’ UTRs of mRNAs [43]. Current literature reports over 550 human microRNAs undergo A-to-I RNA editing [44]. Since there are currently almost 2000 human microRNA sequences annotated on miRBase [45], it seems that ADAR-mediated RNA editing has great implications in microRNA-mediated gene downregulation. Most obviously, RNA editing within the seed region of a microRNA can change that microRNA’s target repertoire [29]. This hypothesis has been recently tested in the context of vascular disease. A-to-I editing of microRNAs increases in vascular tissues in response to ischemia, indicating a key role in post-ischemic neovascularization [46, 47]. Indeed, the edited microRNAs were found to have a completely different set of targets compared to their unedited counterparts promoting the proangiogenic functions of vascular cells in the ischemic/hypoxic context [48]. Deletion of cardiomyocyte-specific *ADAR1* resulted in embryonic lethality, whilst tamoxifen-inducible knockout of *ADAR1* expression in cardiomyocytes resulted in acute heart failure and lethality. RNA-seq data revealed an upregulation of apoptotic genes linked to the observed reduced trabecular compaction, reduced proliferation and increased in the numbers of apoptotic cardiomyocytes [49], attributed to reduced miRNA expression and aggravation of unfolded protein responses (UPR) [50]. Restoring miRNA expression reversed ADAR1-mediated heart failure [50]. A-to-I RNA editing has been shown to have consequences on the fate of microRNAs themselves. Editing at specific nucleotide positions of primary microRNAs (pri-miR) has been shown to induce structural conformation changes which prevent Drosha and/or Dicer from further processing the microRNA [51, 52]. In consequence, this leads to the miRNA being rapidly degraded [53]. Of note, Vik et al*.* showed that editing within sequences that are not binding sites for Drosha and Dicer does not affect microRNA maturation [53]. Interestingly, miR-29b, miR-405 and miR-19 were reported to be downregulated in *Adar2* KO mouse hearts [54]. The presence of cardiomyopathy and cardiac fibrosis, which are known to associate with miR-29b, miR-405 and miR-19 downregulation [55], was also observed in the hearts of these mice. Accordingly, as miR-29b targets filamin B and COL1A2, the expression of both of these genes was upregulated [54]. In another study, AAV9 viral vector-mediated ADAR2 overexpression in cardiomyocytes downregulates miR-34a expression, by interfering with the maturation of its primary transcript. This leads to subsequent upregulation of the miR-34a targets, such as SIRT-1, Cyclin-1 and Bcl2, thereby suggested to reduce in turn the effect of acute myocardial infarction as well as remodelling and doxorubicin-induced cardiotoxicity [56].

Apart from miRNAs, long non-coding RNAs (lncRNAs) shape a great deal of the non-coding genome. LncRNAs play important and multispectral roles in cardiovascular disease [57]. Recently, we reported that ADAR1 targets edit *Neat1* lncRNA, well established for promoting pro-inflammatory gene expression and has also been linked with atherosclerotic cardiovascular disease [15]. RNA editing of *Neat1* lncRNA protects the lncRNA expression by facilitating the recruitment of AUF1, an RNA-binding protein (RBP) with stabilising properties [15].

Splicing may also be affected by RNA editing enzymes. Tang et al*.* identified approximately one hundred high-confidence splicing events that are altered by ADAR1 and/or ADAR2 [58]. Hsiao et al*.* reported approximately 500 native editing sites of ADAR1 within the 3’ splice site acceptor sequences of highly conserved protein-coding genes with very important cellular functions [59]. Such changes account for alternative splicing (exon inclusion/exclusion) or regulation of backsplicing events, which lead to the formation of circRNAs. CircRNAs mostly form from exonic regions of mRNA, where a downstream 5’ splice site of an exon is joined through a reverse complementary match to an upstream 3’ splice site of the same or a different exon, thereby resulting in a circular transcript [60, 61]. CircRNAs hold a key part in cardiovascular biology [62]. Notably, ADARs, which are known suppressors of circRNA biogenesis, have been recently suggested to negatively correlate with significantly circRNAs expression in the transcriptome of heart failure patients [63]. ADAR1-mediated intronic A-to-I RNA editing of smooth muscle cell (SMC) myosin heavy chain (SMMHC) and α-smooth muscle actin (α-SMA) regulates alternative splicing of the target transcripts. Alternative splicing of the edited transcript results in the accumulation of the pre-mRNA and therefore reduced expression of the targets of the mature mRNA, thereby altering smooth muscle cell phenotypes [64]. Therefore, ADAR1-mediated editing controls vascular remodelling in physiology and disease by controlling the phenotypical switch between contractile and synthetic SMC.

Conclusively, the discussed examples underscore the importance of RNA editing for cardiovascular gene expression and, in turn, cardiovascular biology. Although further research is required to determine the relevance of the millions of edited sites, it is not too early to start considering RNA editing from a different prism: its therapeutic exploitation which can be based on the tenets of genome editing recently introduced in the field of cardiovascular disease as well [65]. An increasing number of technologies are currently being developed to harness the therapeutic potential of site-directed RNA editing with application to various monogenic disease treatments in view of the precision medicine era.

## How Can RNA Editing Be Used as a Therapeutic Approach?

### From DNA Editing to RNA Editing

Until recently, precision medicine has been associated mostly with DNA therapeutics [66–68]. The same year Sternberg and Doudna published the later Nobel Prize-winning approach to using the CRISPR/Cas9 DNA editing system as a genetic therapy [69], Stafforst and Schneider published their own study revisiting an older approach, used for the purpose of RNA editing instead [70]. Not much attention was paid to RNA editing at the time, as DNA editing was promising to be a one-and-done solution to all genetic diseases. Indeed, correcting DNA is permanent and all subsequent products of edited genes will be corrected. In contrast, RNA therapies can only target a subset of RNAs and the effected changes are not permanent. However, these advantages have now become clinical (and ethical) concerns, and accordingly, researchers are now taking a second look at RNA as a therapeutic intervention.

The primary concern of such gene therapies remains the long-lasting effects of the permanent alteration of DNA material, which is also subsequently inherited through cell division [71]. This renders RNA editing a clinically safer option. Additionally, there are practical advantages of RNA editing using base deaminases. CRISPR-based RNA editing methods described here do not require a protospacer adjacent motif (PAM), and some Cas13 orthologs also do not require a specific protospacer flanking site (PFS) [72], which means there are no target sequence constraints. RNA editing does not require homology-directed repair (HDR), meaning Cas13 methods can be used in non-dividing cells without special adaptations [73]. Finally, CRISPR-based SDRE methods use nuclease-deficient variants of Cas13, which means they cannot cleave genomic sequences [72, 74].

Several RNA-based therapeutics, some of which we describe here, have already been proven effective in treating various disorders, including cardiovascular disease. Their success fuelled the subsequent and currently ongoing development of site-directed RNA editing treatment approaches, which are at the epicentre of this review and have not yet been studied for cardiovascular treatment.

### Current RNA Therapeutics

Current RNA therapeutic approaches use both coding and non-coding RNA molecules. Coding RNA therapeutics involve the delivery of mRNAs containing instructions for specific proteins to be expressed. The most notable mentions are the two mRNA vaccines developed for the prevention of COVID-19 [75, 76]. There have been other previous attempts to develop similar vaccines in humans for rabies [77], influenza [78], cytomegalovirus [79] and Zika [80]. These drugs have not shown ground-breaking results in clinical trials. However, the success of mRNA vaccines in the prevention of COVID-19 will undoubtedly lead to an increase in interest in developing future RNA drugs.

Non-coding RNA therapeutics are antisense oligonucleotides (ASOs) [81], small interfering siRNAs (siRNAs) [82], microRNAs [83] and aptamers [84]. All are single-stranded RNA molecules which bind to their mRNA targets via complementary base-pairing and subsequently inhibit their translation or facilitate mRNA degradation [85].

Most currently approved RNA therapies are ASOs and have already proven successful in treating human diseases, including cardiovascular disease [85]. The first ever oligonucleotide drug to be approved was Vitravene in 1998 for the treatment of patients with cytomegalovirus retinitis [86]. Subsequently, the approval of Mipomersen by the FDA paved the way for the use of antisense oligonucleotides in the context of cardiovascular disease. Mipomersen is a 20-nucleotide-long antisense oligonucleotide which binds to the apolipoprotein B (ApoB) mRNA and recruits RNase H, which leads to the degradation of the ApoB mRNA. This results in lowered LDL-C levels and has been proven effective in the treatment of homozygous familial hypercholesterolemia [87, 88].

Furthermore, inclisiran is a long-acting RNAi therapeutic agent which targets and inhibits PCSK9 synthesis, thereby lowering blood LDL-particle concentrations [89, 90]. Inclisiran has been recently approved (1 September 2021) by the National Institute for Health and Care Excellence for patients with hypercholesterolaemia or mixed dyslipidaemia who have already had a cardiovascular event such as a heart attack or stroke.

### RNA Editing as a Potential Therapeutic Method: Perspectives on Heart Disease Treatment

Existing RNA therapeutics are efficient in inhibiting or inducing mRNA expression. However, many genetic disorders, including CVDs, are caused by changes in single nucleotides [91]. Site-directed RNA editing (SDRE) is an emerging therapeutic approach that aims to catalyse single base conversions within mRNA molecules, thereby correcting pathological point mutations that occur at the DNA level [92]. The relatively rapid evolution of SDRE methods has been at least in part facilitated by their ability to repurpose and optimise SDRE constructs based on existing therapeutic methods (number and origin of components, size, delivery method). This is crucial, as perfecting these factors can have a significant positive impact on the efficiency and specificity of any drug [93].

**Fig. 2:**
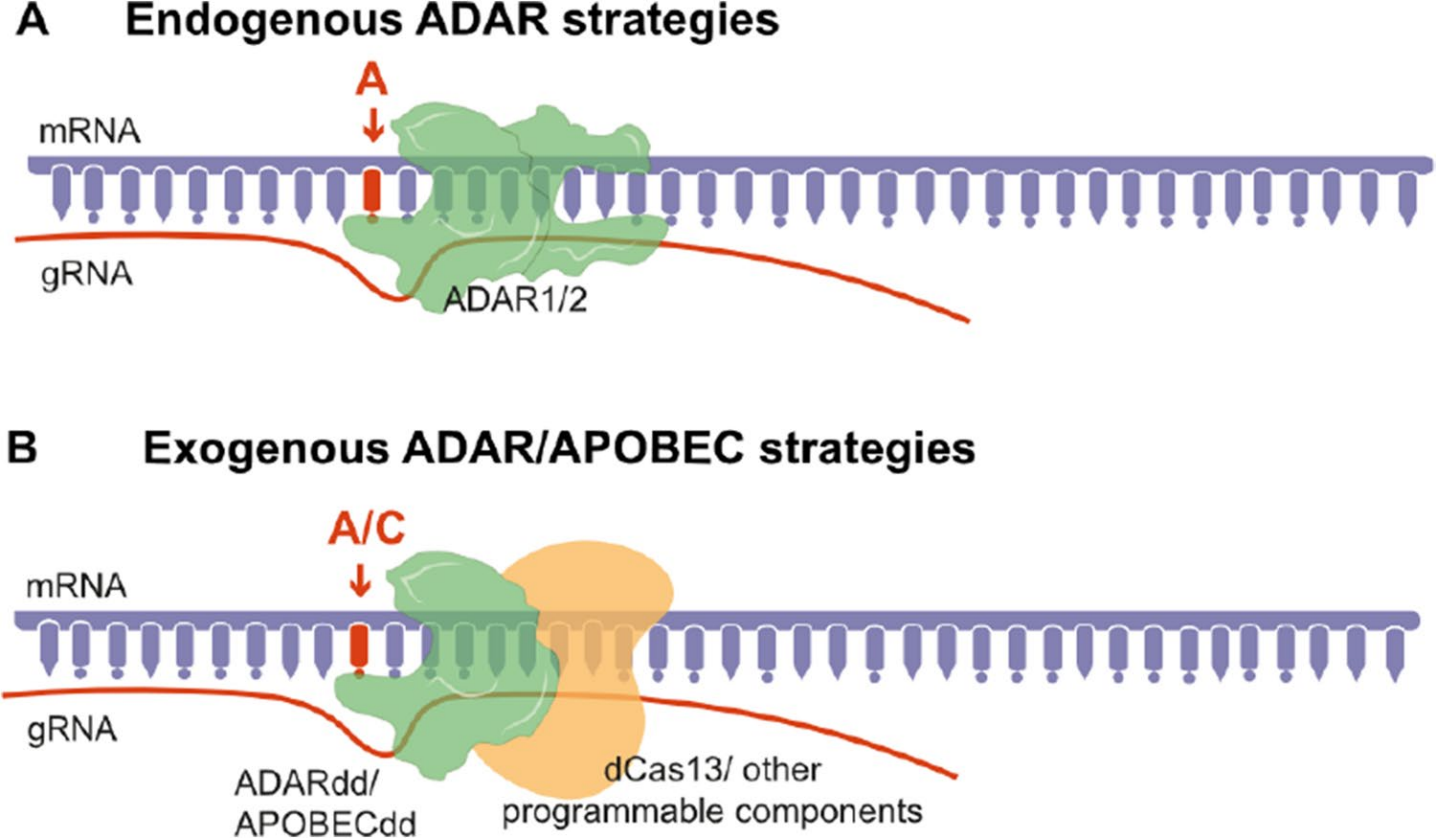
Site-directed RNA editing approaches. **A** Methods using gRNAs to direct endogenous base editors to desired mRNA targets. **B** Methods using gRNAs attached to deaminase domains of base editors guided to the target site using either Cas13 or non-bacterial programmable parts. ADAR, adenosine deaminases acting on RNA; APOBEC, apolipoprotein B mRNA editing catalytic polypeptide-like; gRNA, guide RNA; SDRE, site-directed RNA editing; ADARdd, ADAR deaminase domain; APOBECdd, APOBEC deaminase domain

SDRE methods aim to recruit endogenous molecules or deliver engineered base editing constructs to mRNA targets (Fig. 2A, B). Most of the currently available SDRE strategies use ADARs for A-to-I or C-to-U editing, which is why ADARs are at the centre of this review. To our knowledge, there are only two studies that engineered an SDRE construct using an APOBEC deaminase domain [94, 95], and for one other we are aware of, the authors have expressed the possibility of APOBECs being used as effectors [96].

The key notion considered in SDRE using ADARs is that ADARs target dsRNAs (Fig. 1A). Therefore, to our knowledge, all ADAR SDRE methods developed so far, except for one [95], use guide RNAs (gRNAs). Guide RNAs are single-stranded deoxyribonucleotides, which interact with a target mRNA via Watson–Crick base-pairing, thereby forming a dsRNA structure. A reason why APOBECs may not be currently as widely used for SDRE is possibly akin to the single strandship nature of their substrates (ssRNAs) [97] (Fig. 1B). APOBEBs additionally require binding to specific co-factors, which determine their target specificity/localisation/catalytic activity, whereas ADARs may act independently [97]. APOBECs are also not as widely expressed as ADARs (Fig. 1D), so SDREs aiming to direct endogenously expressed APOBECs to desired targets would not have as many applications.

#### Endogenous ADAR SDREs

Due to their simplicity and quite high efficiency, the most promising SDRE therapeutic methods are currently focused on harnessing the editing abilities of endogenously expressed ADARs, to perform A-to-I editing at mRNA sites that are not their natural targets (Fig. 2A). These methods rely on the use of guide RNAs (gRNAs) that complementarily bind to mRNA targets and form structures which mimic the conformation of natural ADAR targets [81]. Therefore, simply creating dsRNA structures at desired targets can recruit endogenously expressed ADARs [98]. However, mimicking specific substrates of ADARs has proven more efficient. For example, the Gria2 (GluRB) RG stem-loop (GluR2) is a well-known ADAR2 target, with ADAR2-mediated A-to-I editing of GluR2 being essential in brain development [99–101]. This GluR2-ADAR SDRE method uses a 20–40-nucleotide-long gRNA that contains single-stranded region complementary to the mRNA target, as well as a hairpin stem-loop resembling GluR2, which recruits ADAR2’s dsRNA-binding domains [102, 103]. Transient overexpression of ADARs was also shown to increase editing efficiency of this method [102, 103]. Very recently, the Stafforst lab has further improved this gRNA with the addition of a cluster of multiple recruitment sequences, which greatly reduce bystander editing [104].

The stability of the gRNA is a concern, and various modifications can ensure the gRNA is not degraded. For example, a series of chemical modifications have been shown to increase the gRNA’s resistance to enzymatic degradation and editing efficiency (i.e. locked nucleic acids, 2′-O-methylation, phosphorothioate) [105]. Circularisation of the gRNAs has also been proven to increase stability, as circRNAs are intrinsically resistant to cellular exonucleases [106, 107].

#### Engineered SDREs

Although reasonably efficient, SDREs using only gRNAs rely on expression levels of RNA deaminases. Of course, as we have seen in the case of GluR2-ADAR, expression of the gRNA can be accompanied by overexpression of ADARs, for increased editing efficiency [102, 103]. However, the specificity and efficiency of native ADAR SDREs can only be controlled through the gRNA, either by altering its sequence or through introducing chemical modifications [105].

Most engineered SDREs also use gRNAs. However, they include other components for target recognition, as well as either ADAR/APOBEC deaminase domains (Fig. 2B**)**. These additional elements can be individually engineered and modified in order to achieve more specific outcomes. Therefore, these methods are more programmable [108]. For example, several engineered SDREs use inactive Cas13 variants for site recognition. In contrast with Cas9 and Cas12 which possess DNA-binding activity, Cas13 is a type VI CRISPR-associated RNA-guided ribonuclease with RNA-interfering activity [109]. Such methods have been developed for both A-to-I [72, 110, 111] and C-to-U [72, 94, 112] SDRE.

These Cas13 RNA editing systems have proven quite effective. However, they raise two main concerns: cell toxicity due to the bacterial origin of Cas13 and difficulty in vivo delivery due to the large size of the SDRE constructs. However, there has been conflicting evidence regarding the former, suggesting different cell types, organisms and methods of administration may play a part in the level of toxicity of Cas13 [113, 114]. To overcome both potential issues, a smaller but similar system has been developed, which can be engineered entirely out of human parts and has been successfully used for A-to-I SDRE [96, 101]. The authors also describe its potential to be engineered with other effectors, not just ADARs, and it may be used to add/remove a variety of RNA modifications [96].

Research also suggests that endogenous RNAs are competing with engineered gRNAs for binding to the effector proteins used as part of the SDRE constructs, including Cas13 [115]. Therefore, the use of gRNAs might reduce editing efficiency. SDRE systems that use programmable RNA-binding motifs for target recognition instead of gRNAs might be the solution to this issue [95, 116]. Specifically, the programmable RNA-binding motif of the human Pumilio and FBF homology (PUF) proteins has been recently engineered into a novel SDRE system called REWIRE [95, 117]. Fused to an ADAR1/2dd or A3Add, REWIRE achieved high-efficiency A-to-I and C-to-U editing, respectively.

Currently, there are no SDRE clinical trials to our knowledge. However, during a recent investigation, a system that achieves 50% A-to-I editing in vivo, in non-human primates, using a gRNA similar to the GluR2-ADAR method, described above, with no bystander editing, has been developed [118]. Injection of a gRNA in mice resulted in a site-specific RNA editing of respectively 5% and 10% [104].

The most significant limitation for developing SDRE therapies is probably determining target mutations that could most significantly benefit from being corrected. Some SDRE methods do show multiplexing capabilities and can therefore be engineered to edit multiple residues by co-transfection of multiple gRNAs [101, 108]. However, since SDRE methods are in their clinical infancy, focusing on monogenic disorders at this stage is desirable.

Recently, single nucleotide loss of function *PCSK9* A > G mutations have been achieved in non-human primates, through the delivery of DNA base editors in vivo, via a CRISPR editing system [119]*.* The results presented by Musunuru et al*.* show around 60% *PCSK9* suppression in the liver, which is significant in reducing LDL cholesterol and improving the familial hypercholesterolaemia phenotype caused by gain-of-function PCSK9 variants [120]. The DNA A > G editor used by Musunuru et al*.* was engineered [121]. Intriguingly, ADAR1 is ubiquitously expressed [122], including in the liver [123] (Fig. 1D). Therefore, gRNAs can be used to recruit it to the same A residue within the *PCSK9* mRNA and perform SDRE. Since the GluR2-ADAR method has already been tested in non-human primates as well, where it showed 50% editing efficiency, and since this method would only require the engineering of a gRNA, it is certainly worth being tested as a potential therapeutic approach.

## Conclusion

Cardiovascular disease is the leading cause of death worldwide. Therefore, there is an imminent demand to discover therapies able to improve the quality of patients’ life and lower mortality, which remains high despite the recent advances. SDRE represents a novel therapeutic approach that has been proven effective in correcting point mutations preclinically. However, the clinical applications of these methods have not yet been explored in the context of any disease to date. Point mutations are the most common cause of hereditary disease and can affect the expression, stability, structure and function of molecules that are crucial to the development and tissue homeostasis.

SDRE is an emerging therapeutic approach that aims to reverse disease-causing single nucleotide variations with great precision. Unlike DNA editing, RNA editing offers the ability to manipulate genetic information in a tuneable and reversible manner, making it a very attractive approach. In this review, we described the current site-directed RNA editing methods for A-to-I(G) and C-to-U editing using adenosine and/or cytidine RNA deaminases as well as present comparisons between SDRE and other precision medicine approaches, such as DNA editing and RNA inhibition.

Manipulation of adenosine and cytidine deaminases is at the epicentre for the development of site-directed RNA editing. Selective targeting is achieved through Watson–Crick base-pairing between the editing system (through the gRNA in all presented examples except for REWIRE) and the RNA target. The wide variety of SDRE methods allows for applications in various cellular and tissue models, and they can either direct endogenous editors to desired targets or deliver engineered constructs to target mRNAs. Currently, clinical interest is mostly invested in the former type of SDRE, due to its relative simplicity regarding delivery and synthesis, compared to the rather complex engineered constructs of methods using CRISPR, for example, which contain multiple parts and are quite large.

Further non-site-specific therapies are also currently underway in development or clinical trials. For example, pharmaceutical inhibitors against RNA modifying enzymes, including ADAR1, are being developed and scheduled to enter the phase I clinical trial already this year [124]. Although such advancements are fascinating, we believe that given the multispectral role of RNA editing enzymes with regard to the regulation of cardiovascular pathophysiology, as summarised in the present review, such agents need further consolidation before they are promiscuously targeted.

RNA therapeutics are no news to the era of precision medicine. Antisense oligonucleotides have been approved for treating disease as early as 1998, and there are a number of drugs that are currently being used for cardiovascular disease as well. SDRE strategies are anticipated to make subtle but decisive changes in mRNA sequences adequate to reverse the disease course. So far, such therapeutic methods have not been established for the treatment of cardiovascular disease. Nevertheless, our knowledge in terms of synthesis and delivery of oligonucleotides for the purpose of targeting RNA can be applied to site-directed RNA editing methods described in this review. Therefore, site-directed RNA editing approaches are deemed to feature as novel applications replacing existing “not very precise” therapeutic strategies in the near future.

## Data Availability

Not applicable to this study.

## Code Availability

Not applicable to this study.
